# Health care reform and stroke prognosis in low-income Chinese populations from 1992 to 2018

**DOI:** 10.7189/jogh.11.08002

**Published:** 2021-04-17

**Authors:** Jie Liu, Qiuxing Lin, Ying Gao, Xinxin Zhang, Dongwang Qi, Conglin Wang, Jun Tu, Yaogang Wang, Xianjia Ning, Jinghua Wang

**Affiliations:** 1 Department of Neurology, Tianjin Medical University General Hospital, Tianjin, China; 2 Laboratory of Epidemiology, Tianjin Neurological Institute, Tianjin, China; 3 Tianjin Neurological Institute, Key Laboratory of Post-Neuroinjury Neuro-repair and Regeneration in Central Nervous System, Ministry of Education and Tianjin City, Tianjin, China; 4 Health Management Centre, Tianjin Medical University General Hospital, Tianjin, China; 5 Department of Endocrinology and Metabolism, Tianjin Medical University General Hospital, Tianjin, China; 6 Department of Neurology, Yiwu Central Hospital, Yiwu, Zhejiang Province, China; 7 Department of Geriatrics, Tianjin Medical University General Hospital, Tianjin, China; 8 School of Public Health, Tianjin Medical University, Tianjin, China

## Abstract

**Background:**

To assess the impact of the health care reform on stroke prognoses among low-income Chinese residents.

**Methods:**

Stroke events and all-cause deaths were registered during 1992-2018 in Tianjin, China. Trends in stroke management and prognoses were compared during the study periods1992-2008 and 2009-2018.

**Results:**

A total of 1462 patients were diagnosed with first-ever stroke during the study periods. For patients aged ≥45 years, the rates of neuroimaging-based diagnoses and hospitalization were greater in 2009-2018 than in 1992-2008, regardless of patient sex or stroke type. Overall, the one-year case fatality rate was significantly lower in 2009-2018 than in the earlier period; the case fatality rate for women aged ≥65 years decreased by 30.0%. Between both periods, the stroke recurrence rate increased 1.9-fold, including a 2.5-fold increase in men (all *P* < 0.05). During the 2009-2018 period, the one-year case fatality rate was higher among elderly male patients not using medical insurance than among those using it (32.8% vs 20.7%; *P* = 0.050). After 2009, a significant decline in the recurrence rate (*P* = 0.001) and a significant increase in the hospitalization rate (*P* = 0.004) were observed in the interrupted time-series analysis.

**Conclusions:**

These findings suggest that the implementation of universal medical insurance for residents in urban and rural China played a major role in improving the prognoses of low-income, rural, first-ever stroke patients, especially for elderly (≥65 years old) residents. However, elderly male patients not using medical insurance benefits had a high case fatality rate. Thus, restructuring of the government medical insurance policy to facilitate its use by low-income, rural residents is crucial for reducing the stroke burden in China.

Government underfunding and a dearth of health insurance coverage induced widespread public discontent with the limited access and prohibitive costs associated with China’s health care system. In April 2009, China unveiled a huge and complex health reform plan that pledged to provide all citizens, by 2020, with equal access to reasonable-quality, basic health care services and with financial risk protection [1]. Over the past decade, China has made substantial progress towards this goal, especially for people of a lower socioeconomic status [2]. Over this period, government health care expenditures rose from CN¥359 billion (5.7% of total government spending) in 2008 to CN¥1.52 trillion (7.5% of total government spending) in 2017, comprising 1.1% and 1.8% of the gross domestic product, respectively [3]. Moreover, by 2013, insurance coverage was provided to >95% of the country’s population [4]. However, some gaps remain, including control of non-communicable diseases.

Stroke remains the second leading cause of death worldwide [5] and is the leading cause of death and disability adjusted life years (DALYs) in China [6]. However, the age-standardized DALYs for stroke decreased by 33.1% per 100 000 population during the 1990-2017 period. Although stroke incidence and mortality have remained relatively stable, in China, their prevalences continue to increase [7]. With more than 2 million new strokes occurring each year, this disease accounts for the highest number of DALYs among all diseases, in China [6]. Nevertheless, according to the national Healthcare Access and Quality Access index, stroke placed second lowest among 32 diseases or conditions from which death is preventable. This ranking indicated there were inadequate social and medical investments in stroke care, compared with other diseases [8]. These key findings demonstrated that the various strategies for the prevention and control of this disease have not been effectively implemented, in China [7].

The availability of stroke care varies across the country and is especially uneven in rural areas, despite improvements in overall health services [7]. Although standardized secondary prevention can effectively reduce stroke mortality and recurrence, medicines for the secondary prevention of strokes are unavailable and unaffordable for low-income residents. This results in a very low proportion of these individuals using such medicines [9,10]. Our previous study reported that the overall incidence of first-ever strokes in rural China increased by 6.5%, annually, between 1992 and 2012 [11], with the stroke burden focused on young and middle-aged adults [12]. However, data regarding the effectiveness of the national health care reforms at improving stroke prognoses are scarce, especially among low-income populations. Thus, we aimed to assess the effectiveness of 10 years of health care reform on improving stroke prognoses in a low-income population, in China, between 1992 and 2018.

## METHODS

### Study population

The study participants were originally recruited, beginning in 1985, for the Tianjin Brain Study, which was a population-based, stoke surveillance study in a township in Tianjin, China. All patients who experienced first-ever strokes between 1992 and 2018 were included in this population-based prospective study. The year 1992 was chosen as the starting year because neuroimaging procedures (such as computerized tomography) were not available in Tianjin before that year.

The demographic characteristics of the study population were previously described [11,13,14]. Briefly, the study enrolled the residents of 18 administrative villages, 95% of whom were farmers with relatively low levels of income and education. The main source of income was grain production, and the residents had an annual per capita income of<$100 in 1991 and<$1000 in 2010 [4]. In 1991, the illiteracy rate of this population, aged 35-74 years, was 30% for men and 40% for women [15]. Few of these residents were covered by the national medical insurance before 2009.

This study was approved by the ethics committee of Tianjin Medical University General Hospital; written informed consent was obtained from each participant during recruitment.

### Medical insurance information

In China, there are two types of medical insurance: the formal sector employee medical insurance scheme and the resident medical insurance scheme. Tianjin's urban and rural residents’ medical insurance reached universal coverage after the 2009 health care reform [1,16]; before that, medical insurance was scarce in rural areas.

The medical insurance threshold fee is defined as the boundary value for reimbursement of minimum medical expenses. Residents can receive reimbursement for hospitalization expenses and outpatient (emergency) expenses, after deducting the threshold fee. Inpatient expenses are reimbursed at 60%-80% and outpatient (emergency) medical expenses are reimbursed at 50% [17]. In the present study, we used 2008 (immediately before the health care reform) as the time boundary for comparing the prognoses of first-ever stroke patients before and after health care reform.

### Definitions

Stroke management was categorized using the rates of neuroimaging-based diagnoses and hospitalization. Neuroimaging-based diagnosis was defined as patients whose first-ever strokes were diagnosed using computed tomography or magnetic resonance imaging. The rate of neuroimaging-based diagnoses was calculated using the number of patients whose strokes were diagnosed by neuroimaging divided by the number of all first-ever stroke patients. Hospitalization was defined as admission to a county central hospital or to a tertiary-care hospital, and the hospitalization rate was calculated using the number of hospitalized stroke patients divided by the number of all first-ever stroke patients. On the other hand, stroke prognosis was categorized using the case fatality and recurrence rates. A case fatality was defined as death within one year of a first-ever stroke onset; the rate was calculated by dividing the number of stroke patients who died within 30 days of stroke onset by the number of all first-ever stroke patients. Recurrence was defined as a new stroke event occurring >30 days after a first-ever stroke; the rate was calculated by dividing the number of recurrent stroke patients by the number of all first-ever stroke patients.

A first-ever stroke was defined as the first occurrence (no prior history of stroke in patient medical records) of rapidly developing signs of focal neurological disturbances of presumed vascular etiology lasting >24 hours [17]. Stroke events included ischemic stroke (IS), hemorrhagic stroke, and undetermined stroke in this study. IS was defined as a thrombotic brain infarction, cardioembolic stroke, or lacunar infarct; hemorrhagic stroke was defined as intracerebral hemorrhage or subarachnoid hemorrhage; undetermined strokes were defined as strokes that could not be classified into the previously defined types.

All stroke events were full clinical strokes, diagnosed according to pre-established criteria for clinical features and imaging evidence. This definition excluded transient ischemic attacks and silent strokes (those diagnosed using imaging only), but stroke patients with histories of transient ischemic attacks were regarded as suffering incident events. All patients diagnosed using neuroimaging underwent either computed tomography or magnetic resonance imaging at the county central or tertiary care hospital within 24 hours of stroke onset.

### Stroke surveillance and quality control

A stroke surveillance network was established in 1985 [13]; briefly, stroke events were confirmed using the following procedures. First, locally licensed village doctors reported the events to the community hospital within 24 hours of onset (main source of incidence monitoring). Second, for outpatients, community hospital physicians visited the patients’ homes to confirm the events within 72 hours of onset; for inpatients, physicians acquired the patients’ medical records after they were discharged from the hospitals. Confirmed events were reported monthly to neurologists at Tianjin Medical University. Finally, neurologists confirmed all reported events through a door-to-door survey. Another source of incident stroke data was the all-cause death registry, which supplemented missing stroke events. During follow-up, local doctors reported patient outcomes within the first year of a first-ever stroke. The screening of stroke patients in this study covered all permanent residents in Tianjin.

The senior neurologists at Tianjin Medical University General Hospital trained the local area doctors, annually, to ensure accurate reporting of stroke events. The Quality Control Group conducted annual omission surveys that involved comparing multiple overlapping sources, including hospital admission registers, local death registers, and interviews with the relatives of patients, to reduce the number of missing events.

Patient data, including name, sex, age, and years of formal education, were collected through face-to face interviews with patients or their relatives and were conducted by well-trained epidemiology professionals.

### Statistical analysis

Because the rural medical insurance scheme has only been in place since 2009, first-ever strokes and their outcomes were analyzed over two separate periods, 1992-2008 and 2009-2018. The data were analyzed for both men and women after classifying the patients into three age groups (<45, 45-64, ≥65 years). Continuous variables are expressed as means (standard deviations), and categorical variables are expressed as numbers (frequencies). Differences between the two study periods were compared using Student’s *t-*test for continuous variables and the χ^2^ test for dichotomous variables. Statistical significance was defined at *P* < 0.05. The interrupted time-series analysis was performed to assess the impact of health reform on the management and prognosis of stroke. The period 1992-2008 was defined as the period before health reform, and 2009-2018 was defined as the period after health reform. The slope of the trend was presented as the impact of health reform on the management and prognosis of stroke. SPSS for Windows^®^ (version 19.0, SPSS, Chicago, IL, USA) was used to conduct the analyses.

## RESULTS

A total of 1462 patients (58.7% males) experienced first-ever strokes between January 1, 1992, and December 31, 2018. In these patients, the mean age of onset was 66.85 years, and53.2% of patients were ≥65 years old. A larger percentage of patients were aged 45-64 years during the 2009-2018 (47.7%) period than during the earlier period (35.9%; *P* < 0.001). ISs accounted for 77.6% of the overall stroke events, with a significant increase in the proportion of IS events between the later (81.2%) and earlier (73.4%, *P* < 0.001) periods (**Table 1**).

**Table 1:** Demographical characteristics of all patients by study periods

Category	1992-2018	1992-2008	2009-2018	*P*-value
Case, n (%)	1462 (100.0)	668 (45.7)	794 (54.3)	
Gender, n (%):				0.524
Men	858 (58.7)	398 (59.6)	460 (57.9)	
Women	604 (41.3)	270 (40.4)	334 (42.1)	
Age, years, means (SD)	66.85 (11.89)	66.48 (11.70)	65.31 (12.03)	0.62
Age group (years), n (%):				<0.001
<45	65 (4.4)	30 (4.5)	35 (4.4)	
45-64	619 (42.3)	240 (35.9)	379 (47.7)	
≥65	778 (53.2)	398 (59.6)	380 (47.9)	
Education, years, means (SD)	3.50 (3.46)	2.19 (2.99)	4.59 (3.46)	<0.001
Stroke subtypes, n (%):				<0.001
Ischemic stroke	1135 (77.6)	490 (73.4)	645 (81.2)	
Hemorrhagic stroke	271 (18.5)	136 (20.4)	135 (17.0)	
Unknown	56 (3.8)	42 (6.3)	14 (1.8)	

SD – standard deviation

**Table 2** shows that there were increases in the proportions of patients diagnosed using neuroimaging and who were hospitalized during the 2009-2018 period than during the earlier period, for all strokes (*P* < 0.001), with similar trends observed for both men and women. The rate of neuroimaging-based diagnoses, however, remained relatively constant for patients with hemorrhagic strokes (*P* > 0.05).

**Table 2:** Management of first-ever stroke by gender and stroke types during 1992 to 2018

Items	Total			Men			Women		
**1992-2018**	**1992-2008**	**2009-2018**	**1992-2018**	**1992-2008**	**2009-2018**	**1992-2018**	**1992-2008**	**2009-2018**
Stroke, n (%):									
Diagnosis use CT/MRI	1149 (78.6)	442 (66.2)	707 (89.0)*	672 (78.3)	268 (67.3)	404 (87.8)*	477 (79.0)	174 (64.4)	303 (90.7)*
Hospitalized	483 (33.0)	84 (12.6)	399 (50.3)*	280 (32.6)	47 (11.8)	233 (50.7)*	203 (33.6)	37 (13.7)	166 (49.7)*
**Ischamic stroke, n (%):**									
Diagnosis use CT/MRI	896 (78.9)	320 (65.3)	576 (89.3)*	519 (78.2)	195 (66.3)	324 (87.6)*	377 (80.0)	125 (63.8)	252 (91.6)*
Hospitalized	368 (32.4)	54 (11.0)	314 (48.7)*	213 (32.1)	32 (10.9)	181 (48.9)*	155 (32.9)	22 (11.2)	133 (48.4)*
Hemorrhagic stroke, n (%):									
Diagnosis use CT/MRI	253 (93.4)	122 (89.7)	131 (97.0)*	153 (95.0)	73 (92.4)	80 (97.6)	100 (90.9)	49 (86.0)	51 (96.2)
Hospitalized	115 (42.4)	30 (22.1)	85 (63.0)*	67 (41.6)	15 (19.0)	52 (63.4)*	48 (43.6)	15 (26.3)	33 (62.3)*

*Indicates *P* < 0.05 compared between 1992-2008 and 2009-2018.

Moreover, among patients <45 years old, the hospitalization rates were higher for men and the total patient population for all strokes during the 2009-2018 period than during the 1992-2008; women did not show a similar trend. For patients ≥45 years old, the hospitalization rate during the 2009-2018 period was higher than during the 1992-2008 period, regardless of sex or stroke type. The percentage of patients (≥45 years old) diagnosed with ISs (and total strokes), using neuroimaging, was significantly higher during the 1992-2008 period than during the later period (**Table 3**).

**Table 3:** Management of first-ever stroke by age, gender, and stroke types during 1992 to 2018

Items	<45 years			45-64 years			≥65 years		
**1992-2008**	**2009-2018**	***P*-value**	**1992-2008**	**2009-2018**	***P*-value**	**1992-2008**	**2009-2018**	***P*-value**
**Stroke**										
Men:										
Diagnosis use CT/MRI	12 (85.7)	18 (100.0)	0.098	118 (84.9)	231 (98.7)	<0.001	138 (56.3)	155 (74.5)	<0.001
Hospitalized	1 (7.1)	13 (72.2)	<0.001	32 (23.0)	132 (56.4)	<0.001	14 (5.7)	88 (42.3)	<0.001
Women:										
Diagnosis use CT/MRI	15 (93.8)	17 (100.0)	0.295	88 (87.1)	142 (97.9)	0.001	71 (46.4)	144 (83.7)	<0.001
Hospitalized	6 (37.5)	7 (41.2)	0.829	20 (19.8)	84 (57.9)	<0.001	11 (7.2)	75 (43.6)	<0.001
Total:										
Diagnosis use CT/MRI	27 (90.0)	35 (100.0)	0.055	206 (85.8)	373 (98.4)	<0.001	209 (52.5)	299 (78.7)	<0.001
Hospitalized	7 (23.3)	20 (57.1)	0.006	52 (21.7)	216 (57.0)	<0.001	25 (6.3)	163 (42.9)	<0.001
**Ischamic stroke**										
Men:										
Diagnosis use CT/MRI	9 (90.0)	8 (100.0)	0.357	87 (82.1)	181 (98.9)	<0.001	99 (55.6)	135 (75.4)	<0.001
Hospitalized	1 (10.0)	4 (50.0)	0.060	22 (20.8)	103 (56.3)	<0.001	9 (5.1)	74 (41.3)	<0.001
Women:										
Diagnosis use CT/MRI	10 (90.9)	9 (100.0)	0.353	62 (88.6)	117 (97.5)	0.011	53 (46.1)	126 (86.3)	<0.001
Hospitalized	4 (36.4)	4 (44.4)	0.714	11 (15.7)	66 (55.0)	<0.001	7 (6.1)	63 (43.2)	<0.001
Total:										
Diagnosis use CT/MRI	19 (90.5)	17 (100.0)	0.191	149 (84.7)	298 (98.3)	<0.001	152 (51.9)	261 (80.3)	<0.001
Hospitalized	5 (23.8)	8 (47.1)	0.133	33 (18.8)	169 (55.8)	<0.001	16 (5.5)	137 (42.2)	<0.001
**Hemorrhagic stroke**										
Men:										
Diagnosis use CT/MRI	3 (100.0)	10 (100.0)	-	31 (100.0)	50 (98.0)	0.433	39 (86.7)	20 (95.2)	0.292
Hospitalized	0 (0.0)	9 (90.0)	0.003	10 (32.3)	29 (56.9)	0.031	5 (11.1)	14 (66.7)	<0.001
Women:										
Diagnosis use CT/MRI	5 (100.0)	8 (100.0)	-	26 (89.7)	25 (100.0)	0.098	18 (78.3)	18 (90.0)	0.298
Hospitalized	2 (40.0)	3 (37.5)	0.928	9 (31.0)	18 (72.0)	0.003	4 (17.4)	12 (80.0)	0.004
Total:										
Diagnosis use CT/MRI	8 (100.0)	18 (100.0)	-	57 (95.0)	75 (98.7)	0.207	57 (83.8)	38 (92.7)	0.181
Hospitalized	2 (25.0)	12 (66.7)	0.049	19 (31.7)	47 (61.8)	<0.001	9 (13.2)	26 (63.4)	<0.001

For all patients, the one-year case fatality rate for first-ever strokes in 2009-2018 decreased by 27.8%, overall, compared with 1992-2008 (*P* = 0.002). This included a 23.4% decrease among men (*P* = 0.043) and a 34.2% decrease among women (*P* = 0.012). However, the recurrence rate among men was 1.3-fold higher during the 2009-2018 period, compared with the 1992-2008 period (*P* = 0.006). There were no significant changes in the one-year outcomes, across stroke types, between the study periods (**Table 4**).

**Table 4:** Outcomes within one year after first-ever stroke by gender, and stroke types during 1992 to 2018

Outcomes	Total	1992-2008	2009-2018	*P*-value
**Stroke**				
Case fatality:				
Men	183 (21.3)	97 (24.4)	86 (18.7)	0.043
Women	116 (19.2)	64 (23.7)	52 (15.6)	0.012
Total	299 (20.5)	161 (24.1)	138 (17.4)	0.002
Recurrence:				
Men	48 (5.6)	13 (3.3)	35 (7.6)	0.006
Women	24 (4.0)	7 (2.6)	17 (5.1)	0.118
Total	72 (4.9)	20 (3.0)	52 (6.5)	0.002
**Ischemic stroke**				
Case fatality:				
Men	86 (13.0)	36 (12.2)	50 (13.5)	0.629
Women	42 (8.9)	19 (9.7)	23 (8.4)	0.618
Total	128 (11.3)	55 (11.2)	73 (11.3)	0.961
Recurrence:				
Men	42 (6.3)	11 (3.7)	31 (8.4)	0.015
Women	19 (4.0)	5 (2.6)	14 (5.1)	0.167
Total	61 (5.4)	16 (3.3)	45 (7.0)	0.006
**Hemorrhagic stroke**				
Case fatality:				
Men	66 (41.0)	37 (46.8)	29 (35.4)	0.139
Women	51 (46.4)	28 (49.1)	23 (43.4)	0.547
Total	117 (43.2)	65 (47.8)	52 (38.5)	0.123
Recurrence:				
Men	6 (3.7)	2 (2.5)	4 (4.9)	0.432
Women	5 (4.5)	2 (3.5)	3 (5.7)	0.588
Total	11 (4.1)	4 (2.9)	7 (5.2)	0.349
**Undetermined strokes**				
Case fatality:				
Men	31 (93.9)	24 (96.0)	7 (87.5)	0.380
Women	23 (100)	17 (100)	6 (100)	-
Total	54 (96.4)	41 (97.6)	13 (92.9)	0.406
Recurrence:				
Men	–	–	–	–
Women	–	–	–	–
Total	–	–	–	–

**Table 5** indicates the absence of significant changes in case fatality or recurrence rates, between the two periods, for patients aged <65 years, regardless of stroke type. However, the overall one-year case fatality rate for all stroke patients aged ≥65 years during the 2009-2018 period was lower than that during the 1992-2008 period (25.5% vs 33.7%; *P* = 0.013); the case fatality rate for women ≥65 years old decreased by 30.0% (23.3% vs 33.3%; *P* = 0.043). Between the two study periods, the overall stroke recurrence rate increased 1.9-fold (*P* = 0.001), and the recurrence rate among male patients aged ≥65 years increased 2.5-fold (*P* = 0.010). For hemorrhagic stroke patients aged ≥65 years, the overall case fatality rate decreased by 44.5% (*P* = 0.042), including by 30.0% among men (*P* = 0.043), between both study periods; the recurrence rate increased significantly among men (*P* = 0.036). Meanwhile, there were no significant changes in the one-year outcomes in undetermined strokes between both study periods (all *P* > 0.05).

**Table 5:** Outcomes within one year after first-ever stroke by age, gender, and stroke types during 1992 to 2018

Outcomes	<45 years			45-64 years			≥65 years		
**1992-2008**	**2009-2018**	***P*-value**	**1992-2008**	**2009-2018**	***P*-value**	**1992-2008**	**2009-2018**	***P*-value**
**Stroke**											
Case fatality:											
Man	1 (7.1)	3 (16.7)	0.419	13 (9.4)	26 (11.1)	0.591	83 (33.9)	57 (27.4)	0.137		
Woman	2 (12.5)	2 (11.8)	0.948	11 (10.9)	10 (6.9)	0.270	51 (33.3)	40 (23.3)	0.043		
Total	3 (10.0)	5 (14.3)	0.600	24 (10.0)	36 (9.5)	0.837	134 (33.7)	97 (25.5)	0.013		
Recurrence:											
Man	0	0	–	6 (4.3)	14 (6.0)	0.490	7 (2.9)	21 (10.1)	0.010		
Woman	0	0	–	3 (3.0)	7 (4.8)	0.468	4 (2.6)	10 (5.8)	0.156		
Total	0	0	–	9 (3.8)	21 (5.5)	0.312	11 (2.8)	31 (8.2)	0.001		
**Ischemic stroke**											
Case fatality:											
Man	0	0	–	1 (0.9)	7 (3.8)	0.150	35 (19.7)	43 (24.0)	0.319		
Woman	0	0	–	1 (1.4)	1 (0.8)	0.698	18 (15.7)	22 (15.1)	0.897		
Total	0	0	–	2 (1.1)	8 (2.6)	0.267	53 (18.1)	65 (20.0)	0.546		
Recurrence:											
Man	0	0	–	4 (3.8)	12 (6.6)	0.319	7 (3.9)	19 (10.6)	0.015		
Woman	0	0	–	2 (2.9)	5 (4.2)	0.644	3 (2.6)	9 (6.2)	0.173		
Total	0	0	–	6 (3.4)	17 (5.6)	0.277	10 (3.4)	28 (8.6)	0.007		
**Hemorrhagic stroke**											
Case fatality:											
Man	0 (0.0)	3 (30.0)	0.279	10 (32.3)	19 (37.3)	0.646	27 (60.0)	7 (33.3)	0.043		
Woman	2 (40.0)	2 (25.0)	0.569	8 (27.6)	9 (36.0)	0.507	18 (78.3)	12 (60.0)	0.193		
Total	2 (25.0)	5 (27.8)	0.883	18 (30.0)	28 (36.8)	0.402	45 (66.2)	19 (46.3)	0.042		
Recurrence:									
Man	0	0	–	2 (6.5)	2 (3.9)	0.606	0 (0.0)	2 (9.5)	0.036		
Woman	0	0	–	28 (96.6)	23 (92.0)	0.467	1 (4.3)	1 (5.0)	0.919		
Total	0	0	–	3 (5.0)	4 (5.3)	0.945	1 (1.5)	3 (7.3)	0.116		
**Undetermined strokes**											
Case fatality:											
Man	1 (100)	0	–	2 (100)	0	–	21 (95.5)	7 (87.5)	0.440	
Woman	-	0	–	2 (100)	0	–	15 (100)	6 (100)	–	
Total	1 (100)	0	–	4 (100)	0	–	36 (97.3)	13 (92.9)	0.466	
Recurrence:											
Man	0	0	–	0	0	–	0	0	–		
Woman	0	0	–	0	0	–	0	0	–		
Total	0	0	–	0	0	–	0	0	–		

Since 2009, more than 95% of the residents in the study population were covered by universal medical insurance. However, only 52% of the stroke patients used their medical insurance; the remaining 48% did not as they could not afford the threshold fee. During the 2009-2018 period, the overall one-year case fatality rate for first-ever strokes was higher among male patients who did not use medical insurance (22.6%) than among those who did (15.2%; *P* = 0.043). A similar trend was observed among patients aged ≥65 years; the case fatality rate was 20.7% for the insured patients and 32.8% for the un-insured patients (*P* = 0.050). However, there were no significant differences in the recurrence rates, regardless of age (**Table 6**).

**Table 6:** Outcomes within one year after first-ever stroke by application of medical insurance during 2009 to 2018

Outcomes	Total	Used insurance	Unused insurance	*P*-value
**All**				
Case number	794 (100)	413 (52.0)	381 (48.0)	–
Case fatality:				
Men	86 (18.7)	37 (15.2)	49 (22.6)	0.043
Women	52 (15.6)	25 (14.8)	27 (16.4)	0.692
Total	138 (17.4)	62 (15.0)	76 (19.9)	0.072
Recurrence:				
Men	35 (7.6)	18 (7.4)	17 (7.8)	0.863
Women	17 (5.1)	10 (5.9)	7 (4.2)	0.486
Total	52 (6.5)	28 (6.8)	24 (6.3)	0.770
**<45 years**				
**Case number**				
Case fatality:				
Men	3 (16.7)	3 (23.1)	0	0.522
Women	2 (11.8)	1 (50.0)	1 (50.0)	1.000
Total	5 (14.3)	4 (19.0)	1 (7.1)	0.627
Recurrence:				
Men	0	0	0	–
Women	0	0	0	–
Total	0	0	0	–
**45-64 years**				
**Case number**				
Case fatality:				
Men	26 (11.1)	15 (10.9)	11 (11.5)	0.888
Women	10 (6.9)	7 (8.3)	3 (4.9)	0.520
Total	36 (9.5)	22 (9.9)	14 (8.9)	0.745
Recurrence:				
Men	14 (6.0)	10 (7.2)	4 (4.2)	0.329
Women	7 (4.8)	3 (3.6)	4 (6.6)	0.454
Total	21 (5.5)	13 (5.9)	8 (5.1)	0.750
**≥65 years**				
**Case number**				
Case fatality:				
Men	57 (27.4)	19 (20.7)	38 (32.8)	0.050
Women	40 (23.3)	17 (22.1)	23 (24.2)	0.742
Total	97 (25.5)	36 (21.3)	61 (28.9)	0.091
Recurrence:				
Men	21 (10.1)	8 (8.7)	13 (11.2)	0.550
Women	10 (5.8)	7 (9.1)	3 (3.2)	0.114
Total	31 (8.2)	15 (8.9)	16 (7.6)	0.647

**Figure 1:**
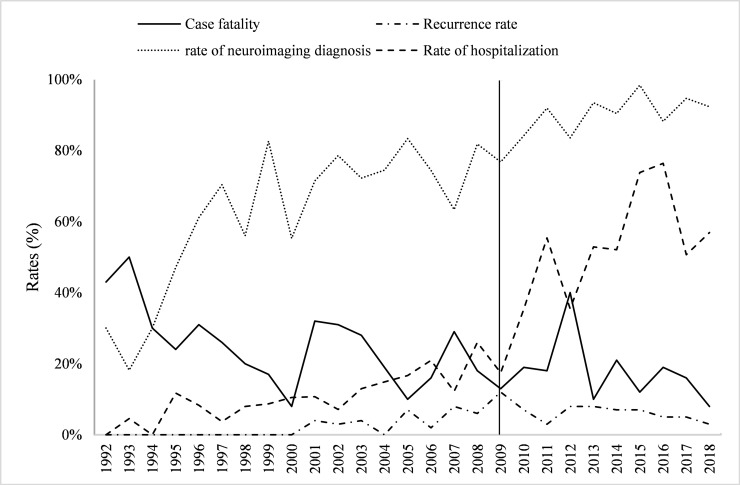
The interrupted time-series analysis of management and prognosis among stroke patients. Figure 1 showed that a significant decline in the recurrence rate (*P* = 0.001) and a significant increase in the hospitalization rate (*P* = 0.004) occurred after 2009. However, no significant differences were observed for trends incase fatality (*P* = 0.702) and diagnosis using neuroimaging (*P* = 0.285).

The interrupted time-series analysis showed that before the implementation of the policy (1992-2008), the case fatality rate decreased, and the slope was -0.012 (95% CI = -0.022, -0.003; *P* = 0.015). After the implementation of the policy (2009-2018), the slope of the case fatality rate was -0.008 (95% CI = -0.031, -0.015; *P* = 0.443). In 1992-2008, the recurrence rate increased, with a slope of 0.004 (95% CI = 0.002, 0.006; *P* < 0.001), while in 2009-2018, the recurrence rate decreased, with a slope of-0.005 (95% CI = -0.011, 0.000; *P* = 0.061). In addition, for hospitalization, the corresponding slopes for both periods were 0.011 (95% CI = 0.007, 0.015; *P* < 0.001) and 0.044 (95% CI = 0.010, 0.077; *P* = 0.017), and for diagnosis using neuroimaging, the corresponding slopes for both periods were 0.031 (95% CI = 0.018, 0.045; *P* < 0.001) and 0.017 (95% CI = 0.002, 0.031; *P* = 0.027). After 2009, a significant decline in the recurrence rate (*P* = 0.001) and a significant increase in the hospitalization rate (*P* = 0.004) occurred. However, no significant differences were observed for trends incase fatality (*P* = 0.702) and diagnosis using neuroimaging (*P* = 0.285; **Figure 1**).

## DISCUSSION

This is the first report describing the association of stroke outcomes with the utilization of medical insurance in a low-income population in rural China following the 2009 national health care reform. The preponderant stroke type was IS, the proportion of which increased significantly from the pre-reform (1992-2008) to the post-reform (2009-2018) period. The proportions of strokes diagnosed using neuroimaging and patients who were hospitalization also significantly increased between the later and earlier periods, especially among patients aged ≥45 years. The increase in hospitalization rate was significantly higher in 2009-2018 than in 1992-2008. For all first-ever stroke patients, the one-year case fatality rate during the 2009-2018 period was lower for both males and females than it was during the earlier period. The trend of overall recurrence rate decreased after 2009, although the recurrence rate in men was1.3-fold higher in 1992-2008 than in2009-2018. Moreover, the overall one-year case fatality rate for patients aged ≥65 years was lower during the later period than during the earlier one; the recurrence rates for all strokes, ISs, and hemorrhagic strokes were higher among men. During the 2009-2018 period, the overall (and male) case fatality rate was higher among non-insured patients than among insured patients; among patients aged ≥65 years, there were 58.5% more case fatalities among the non-insured patients than among the insured patients.

A population-based survey of 480 687 adults, in China, indicated that the stroke incidence has declined in urban areas and risen in rural areas over the past 30 years [18]. Our previous study demonstrated that, in rural China, the IS incidence increased sharply, particularly among middle aged adults, between 1992 and 2012 [19]. However, prior to 2008, the percentage of rural stroke patients undergoing computed tomography examinations was low [20], reflecting the low level of medical spending in rural areas before 2009. However, according to 2019 data from the National Bureau of Statistics of China [21], bed utilization rates in township health centers have increased from 55.8% (2008) to 59.6% (2018) and the personally financed proportion of medical expenses has increased from 7.8% to 10.2%. A study from Gansu, China indicated that both total and out-of-pocket costs have fallen, while actual compensation rates have risen since the implementation of the new rural health insurance payment system [22]. Before 2008, 79% of rural residents and 45% of urban residents did not have health insurance; in 2018, health care insurance covered >95% of China’s citizens [3]. Between 2008 and 2017, outpatient visits increased by 12.1% at tertiary hospitals, by 3.4% at secondary level hospitals, and by 4.4% at primary health care facilities, annually; per outpatient visit charges increased at 6.7%-7.1%, annually [2]. In this study, the proportions of patients diagnosed using neuroimaging and who were hospitalized, were significantly higher during the 2009-2018 period than during the 1992-2008 period, especially among patients ≥45 years old. These findings demonstrated that the health care system has improved the ability of rural residents to pay for medical care; this is especially apparent among middle-aged and elderly patients. Although China's health care reform began only 13 years ago, its achievements are obvious.

The one-year case fatality rate for first-ever stroke patients during the 2009-2018 period was significantly lower than during the 1992-2008 period, especially among female patients aged ≥65 years. In China, health care for women, especially elderly women, was neglected due to their low socioeconomic status. Thus, elderly women were often treated at home or by the local village doctor. The results of this study demonstrate, to a certain extent, that the universal medical insurance, implemented in 2009, has benefited rural residents and decreased the stroke burden.

For most post-stroke patients, secondary prevention medications are now prescribed, beginning during hospitalization; however, poor post-discharge adherence continues to be a problem, especially in rural China [18,19]. A previous study reported that among post-stroke patients, only 10.6%, 10.1%, 7.6%, 2.3%, 18.2%, and 1.4% were regularly taking antiplatelet drugs, β-blockers, angiotensin-converting enzyme inhibitors, diuretics, calcium-channel blockers, and statins, respectively [19]. In one study [18], the use of anti-platelet agents (18.6%) and any of the blood pressure (BP)-lowering drugs (38.2%) by patients with histories of either ischemic heart disease or stroke, in China, were much lower than for patients in similar North American [20] (anti-platelet drugs, 52.2%; BP-lowering medication, 69.2%) and Middle Eastern (anti-platelet drugs, 49.7%; BP-lowering medication, 64.3%) studies. In addition, there were significantly different levels of secondary prevention provided to patients with high socioeconomic status than to those with low socioeconomic status, in China [18]. Part of the reason for the alarmingly low use of secondary prevention medications is the inadequate ability of low-income people to pay for these medications [18,21]. In the present study, although the one-year stroke case fatality rate was remarkably lower during the 2009-2018 period than during the 1992-2008 period, the stroke recurrence rate during the later period was more than twice that during the earlier period, indicating that the secondary prevention of cerebrovascular disease is far from ideal in rural China. Both the poor awareness of the benefits of secondary prevention methods and the inability to pay for medicines, among rural residents, are major causes of the high recurrence rate [22]. However, the downward trend in the recurrence rate of stroke showed that the universal medical insurance in China plays a positive role in stroke recurrence.

Although the Chinese government has implemented a universal medical insurance policy [2], there are still some low-income groups who are unable to pay even the threshold fee. Moreover, stroke recurrence aggravates poverty, forming a vicious circle in low-income populations. As a result, China will continue to experience an enormous health burden due to strokes as >41.48% of the total population lives in rural areas [3].

Many of the health inequities in low- and middle-income countries are related to insurance status. Thus, the inevitable consequence is that serious and costly diseases, like strokes, very often have disastrous financial consequences for the patients and their families, especially for low-income patients in rural areas. Out-of-pocket payments, as a portion of total health care spending, are very high in both China and India [23,24]. According to a 2003 national census, 45% of residents in urban areas and 80% of those in rural areas of China were not covered by public insurance [25]. In April 2009, China unveiled a huge and complex health reform plan designed to provide all citizens with equal access to basic health care by 2020 [1]. However, the coverage provided through this program is very small, in terms of both the service benefit package and financial protection provided [26]. In many parts of China, outpatient services are under-insured or not covered. Inpatient services, if covered, still require patients to bear a number of costs (co-payments, deductibles, or additional fees). For instance, the rural cooperative medical scheme only reimburses about 30% of hospitalization expenses [27]. In many cases, medical assistance programs for poor people simply help them participate in the rural scheme rather than paying more of the treatment costs. As a result, the access that poor people actually have to primary care has not really improved, and financial protection against high medical costs remains very limited [16]. In the present study, the mortality rate was significantly higher among male patients not using medical insurance than among those using it. This was especially true among patients aged ≥65 years who had a mortality rate that was 58.5% higher than for those with medical insurance. Therefore, to effectively reduce the stroke burden, the government will need to formulate health insurance policies that are more in-line with the ability of low-income people to pay for their medical services.

This study has several limitations. First, the study population was from one township in northern China, which is not representative of the overall rural population of China. However, the consistency of the national medical insurance policy allows these results to represent the overall situation in rural China. Second, the number of stroke cases was limited. However, the observation time was 391 748 person-years, which meets the criterion for epidemiological studies in stroke patient populations. Third, early in this study, the low rate of neuroimaging-based diagnoses may have affected the determination of stroke types. Fourth, the study population was characterized as being low-income and poorly educated, with access to poor medical services. Thus, some patients with acute attacks may not have been diagnosed in a timely manner, leading to an underestimation of the recurrence rate.

## CONCLUSIONS

Compared to the 1992-2008 period, the percentages of stroke patients diagnosed using neuroimaging and who were hospitalized were significantly higher during the 2009-2018 period. Further, the stroke case fatality rate was remarkably higher among patients not using medical insurance than among those who used medical insurance; the stroke recurrence rate also showed a downward trend after 2009. Moreover, men without adequate medical insurance benefits, due to their low incomes, demonstrated high mortality rates. These findings suggest that the universal medical insurance for urban and rural residents, in China, has played a major role in improving the management and prognoses of first-ever stroke patients residing in rural, low-income areas of China, especially for patients aged ≥65 years. However, there is a crucial need for the government to restructure its medical insurance policy to facilitate access to medical care by low-income, rural residents to reduce the stroke burden in China, especially for those poverty residents.
